# Small-angle neutron scattering correlation functions of bulk magnetic materials

**DOI:** 10.1107/S1600576715013187

**Published:** 2015-08-29

**Authors:** Denis Mettus, Andreas Michels

**Affiliations:** a Physics and Materials Science Research Unit, University of Luxembourg, 162A Avenue de la Faïencerie, L-1511 Luxembourg, Luxembourg

**Keywords:** small-angle neutron scattering, correlation function, micromagnetics, magnetic materials

## Abstract

On the basis of the continuum theory of micromagnetics, the correlation function of the spin-misalignment small-angle neutron scattering cross section of bulk ferromagnets (*e.g.* elemental polycrystalline ferromagnets, soft and hard magnetic nanocomposites, nanoporous ferromagnets, or magnetic steels) is computed. For such materials, the spin disorder which is related to spatial variations in the saturation magnetization and magnetic anisotropy field results in strong spin-misalignment scattering dΣ_M_/dΩ along the forward direction. When the applied magnetic field is perpendicular to the incoming neutron beam, the characteristics of dΣ_M_/dΩ (*e.g.* the angular anisotropy on a two-dimensional detector or the asymptotic power-law exponent) are determined by the ratio of magnetic anisotropy field strength *H*_p_ to the jump Δ*M* in the saturation magnetization at internal interfaces. Here, the corresponding one- and two-dimensional real-space correlations are analyzed as a function of applied magnetic field, the ratio *H*_p_/Δ*M*, the single-particle form factor and the particle volume fraction. Finally, the theoretical results for the correlation function are compared with experimental data on nanocrystalline cobalt and nickel.

## Abstract 2

On the basis of the continuum theory of micromagnetics, the correlation function of the spin-misalignment small-angle neutron scattering cross section of bulk ferromagnets is computed and discussed.

## 1. Introduction   

Small-angle neutron scattering (SANS) is a very popular method for investigating nanoscale structural and magnetic inhomogeneities in the bulk of materials. In most situations, SANS data are analyzed in reciprocal space, by fitting a particular model to the experimental SANS cross section. An alternative real-space approach to analyzing SANS data is the computation of the (auto)correlation function of the system, for instance by means of the indirect Fourier transformation technique (Glatter, 1977 ▸; Hansen, 2000 ▸; Fritz & Glatter, 2006 ▸; Hansen, 2012 ▸), which has recently been extended to allow for the analysis of two-dimensional small-angle scattering patterns of oriented samples (Fritz-Popovski, 2013 ▸; Fritz-Popovski, 2015 ▸). For dilute, monodisperse and uniform particle–matrix systems, several analytical expressions for the density–density autocorrelation function 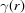 or, likewise, for the distance distribution function 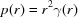 have been derived (see *e.g.* Svergun & Koch, 2003 ▸); this is a well established procedure in small-angle X-ray scattering and in nuclear SANS, *e.g.* in the analysis of polymers (Mortensen & Pedersen, 1993 ▸) or in the study of the formation of magnetic nanocrystals in glass ceramics (Lembke *et al.*, 1999 ▸).

In the context of real-space analysis of scattering data, it is also worth mentioning the recent progress made in the computation of the magnetic pair distribution function (Frandsen *et al.*, 2014 ▸), which is obtained *via* Fourier transformation of the magnetic neutron scattering cross section. This approach permits the analysis of long- and short-range magnetic correlations of a wide range of magnetic structures such as spin-density waves, spin-ice compounds or molecular magnets.

We have recently provided a theory of magnetic SANS of polycrystalline bulk ferromagnets (Honecker & Michels, 2013 ▸), which was successfully employed in order to analyze the magnetic microstructure of iron-based two-phase nanocomposites (Honecker *et al.*, 2013 ▸). In addition to nanocomposites, the theory is also applicable to the study of elemental ferromagnets, nanoporous magnets or ferromagnetic steels; it provides information on the exchange-stiffness constant, as well as on the strength and spatial structure of the magnetic anisotropy and magnetostatic field.

Magnetic SANS of statistically isotropic bulk ferromagnets is, in contrast to nuclear SANS on such structures, highly anisotropic, *i.e.* the magnetic SANS cross section depends not only on the magnitude but also on the orientation of the momentum-transfer vector. The results for the Fourier coefficients of the magnetization (Honecker & Michels, 2013 ▸) demonstrate the unmistakable impact of the magnetodipolar interaction on magnetic SANS. Magnetostatics is essential for understanding the complex magnetic field-dependent angular anisotropies which may be observed on a two-dimensional position-sensitive detector; these anisotropies go beyond the well known ‘
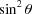
’ anisotropy of magnetic SANS. Furthermore, the classical particle–matrix concept of small-angle scattering is not adapted to the complex magnetic textures that may form inside the bulk of magnetic media [see discussion in the introduction of Michels (2014 ▸)]; for such materials, the continuum theory of micromagnetics (Brown, 1963 ▸) provides the proper theoretical framework for computing the magnetic SANS cross section. It is the purpose of this paper to provide a discussion of the predictions of our micromagnetic SANS theory in real space by calculating the correlation function of the spin-misalignment SANS cross section.

The paper is organized as follows: §2 introduces the model for the magnetic microstructure of bulk ferromagnets, which underlies our magnetic SANS theory; in §3, we summarize the main expressions for the unpolarized magnetic SANS cross section; in §4, we define the correlation function of the spin-misalignment SANS cross section, and we compare its definition with the corresponding result from nuclear SANS theory; §5 details the models for the anisotropy field and longitudinal magnetization Fourier coefficient; in §6, we discuss the results for the correlation functions and correlation lengths, and we provide a comparison with experimental data; §7 summarizes the main findings of this study.

## 2. Model for the magnetic microstructure of bulk ferromagnets   

**Figure 1:**
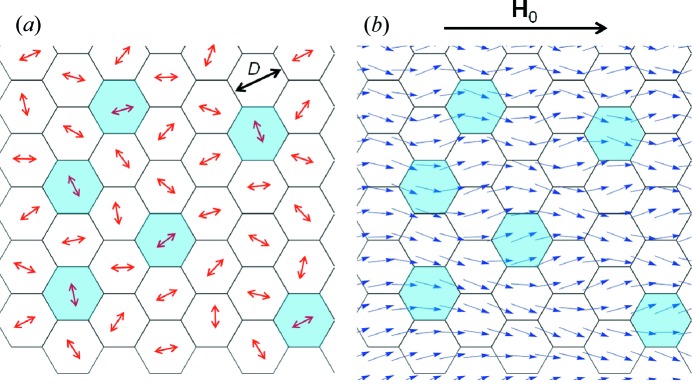
Model for the magnetic microstructure of bulk ferromagnets. (*a*) Sketch of an idealized two-dimensional (nuclear) grain microstructure. The two main sources that cause a perturbation of the magnetic microstructure are identified in our magnetic SANS theory (Honecker & Michels, 2013 ▸) as (i) spatial (random) variations in the direction and/or magnitude of the magnetic anisotropy field and (ii) spatial variations in the magnitude of the saturation magnetization. The characteristic length scales (correlation lengths) over which such variations occur may be related, for example, to the average particle or crystallite size *D*, which for bulk nanomagnets is typically of the order of 10–20 nm. In (*a*), the crystallographic set of easy axes for the magnetization changes randomly at each internal interface (*e.g.* a grain boundary); for simplicity, we have here assumed a uniaxial magnetic anisotropy (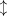). In addition, the magnetic material’s parameters (exchange constant *A*, anisotropy constant *K* and saturation magnetization 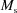) may depend on the position inside the material [which is symbolized by grains (cells) with different color]. (*b*) Superposed (magnetic) spin microstructure in the presence of a strong applied magnetic field 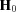. The shown coarse-grained distribution of spins is only qualitative, but suggests the existence of continuously varying nanoscale magnetization profiles, which give rise to a strongly field-dependent magnetic SANS cross section. Note also the absence of sharp interfaces in the magnetic microstructure (*b*), in contrast to the grain microstructure (*a*).

We consider polycrystalline statistically isotropic bulk ferromagnets. Examples of such materials are inert-gas condensed single-phase elemental ferromagnets (Weissmüller *et al.*, 2004 ▸; Löffler *et al.*, 2005 ▸; Michels *et al.*, 2008 ▸, 2009 ▸; Döbrich *et al.*, 2012 ▸), soft magnetic two-phase nanocomposites from the FINEMET (VITROPERM) or NANOPERM family of alloys (Ohnuma *et al.*, 2000 ▸; Heinemann *et al.*, 2000 ▸; Michels *et al.*, 2006 ▸), NdFeB-based permanent magnets (Bick *et al.*, 2013 ▸; Périgo *et al.*, 2015 ▸), and magnetic steels (Coppola *et al.*, 1998 ▸; Bischof *et al.*, 2007 ▸; Michaud *et al.*, 2007 ▸; Alinger *et al.*, 2009 ▸; Bergner *et al.*, 2013 ▸). Fig. 1 ▸(*a*) shows a sketch of the nuclear (grain) microstructure of such a material, and Fig. 1 ▸(*b*) displays qualitatively the magnetic (spin) distribution at a nearly saturating applied magnetic field.

On the basis of the continuum theory of micromagnetics (Brown, 1963 ▸), we have provided (Honecker & Michels, 2013 ▸) a first-order theory for the magnetic spin-misalignment SANS cross section of weakly inhomogeneous bulk ferromagnets, which accounts for spatial variations in the magnetic anisotropy and saturation magnetization. The theory, valid close to magnetic saturation, is based on the solution of the well known balance-of-torques equation, 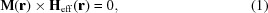
which expresses the fact that at static equilibrium the torque on the magnetization vector field 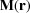 due to an effective magnetic field 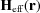 vanishes everywhere inside the material. The effective field 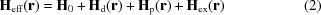
is composed of a uniform applied magnetic field 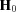, the magnetostatic field 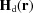, the magnetic anisotropy field 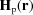 and the exchange field 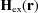. The general solution of equation (1) for the transverse magnetization Fourier coefficients (in the high-field limit) is given in Appendix *A* [App appa]. Metlov & Michels (2015 ▸) extended the first-order theory to second order in the amplitudes of the inhomogeneities (including fluctuations in the exchange interaction), and the corresponding magnetic SANS cross section was computed up to the third order. For the sake of a self-contained presentation, we summarize in §3 the main results for the magnetic SANS cross section of bulk magnetic materials.

## 3. Magnetic SANS theory of bulk ferromagnets – unpolarized neutrons   

**Figure 2:**
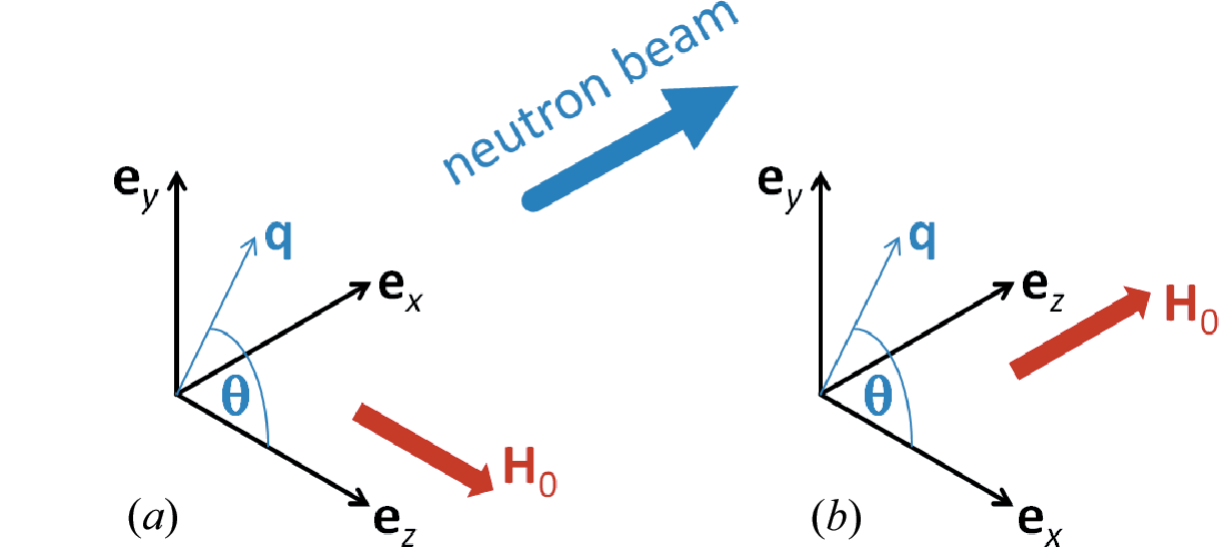
Sketch of the two most often employed scattering geometries in magnetic SANS experiments. (*a*) 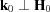; (*b*) 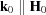. We emphasize that in both geometries the applied-field direction 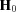 defines the 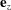 direction of a Cartesian laboratory coordinate system. The angle θ specifies the orientation of the scattering vector on the two-dimensional detector; θ is measured between 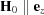 and 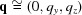 (*a*) and between 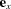 and 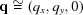 (*b*).

Since the spin-misalignment scattering of bulk ferromagnets is independent of the polarization of the incident neutron beam, it is sufficient to restrict the considerations for the correlation function to the unpolarized cross section. As discussed by Michels (2014 ▸), half-polarized (SANSPOL) experiments on bulk magnetic materials do not provide significantly more information regarding the spin-misalignment SANS than can already be learned from the analysis of unpolarized data; this is because the SANSPOL ‘spin-up’ and ‘spin-down’ cross sections differ essentially only by a nuclear-magnetic interference term 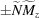, which is usually small and weakly field dependent as compared to the spin-misalignment SANS. In order to demonstrate the main effects, we concentrate in the following on the unpolarized magnetic SANS of bulk ferromagnets in the two scattering geometries that have the applied magnetic field 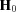 either perpendicular or parallel to the incident neutron-beam direction (see Fig. 2 ▸). The corresponding equations for polarized SANS and, in particular, the spin-flip (POLARIS) equations are given by Honecker *et al.* (2010 ▸) and Michels (2014 ▸).

### 3.1. **k**_0_ ⊥ **H**_0_   

For the scattering geometry where the applied magnetic field 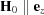 is perpendicular to the wavevector 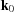 of the incoming neutron beam (compare Fig. 2 ▸ *a*), the elastic unpolarized SANS cross section 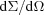 at scattering vector 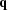 can be written as (Michels, 2014 ▸) 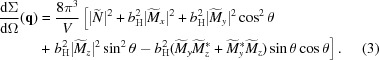


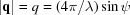
, where ψ is half the scattering angle and λ is the wavelength of the incident radiation, *V* is the scattering volume, 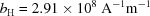 relates the atomic magnetic moment to the Bohr magneton, 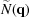 and 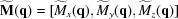 denote, respectively, the Fourier coefficients of the nuclear scattering length density and of the magnetization 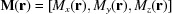, and θ represents the angle between 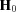 and 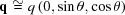; the asterisks (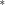) mark the complex conjugate quantity, and the atomic magnetic form factor (in the expression for 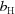) is approximated to unity (forward scattering).

As shown by Honecker & Michels (2013 ▸), near magnetic saturation and for a weakly inhomogeneous bulk ferromagnet, 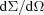 can be evaluated by means of micromagnetic theory. In particular, 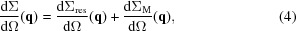
where 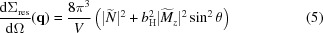
represents the nuclear and magnetic residual SANS cross section, which is measured at complete magnetic saturation (infinite field), and 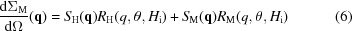
is the spin-misalignment SANS cross section. The magnetic scattering due to transverse spin components, with related Fourier amplitudes 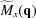 and 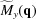, is contained in 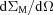, which decomposes into a contribution 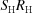 due to perturbing magnetic anisotropy fields and a part 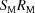 related to magnetostatic fields. The micromagnetic SANS theory considers a uniform exchange interaction and a random distribution of magnetic easy axes, but takes explicitly into account variations in the magnitude of the magnetization [*via* the function 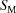, see equation (8) below].

The anisotropy-field scattering function (in units of 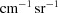) 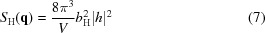
depends on the Fourier coefficient 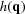 of the magnetic anisotropy field, whereas the scattering function of the longitudinal magnetization (in units of 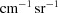) 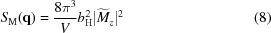
provides information on the magnitude 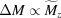 of the magnetization jump at internal (*e.g.* particle–matrix) interfaces. The corresponding (dimensionless) micromagnetic response functions can be expressed as 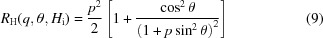
and 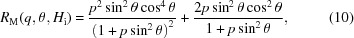
where 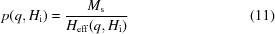
is a (dimensionless) function. The effective magnetic field 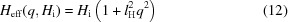
depends on the internal magnetic field 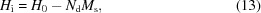
on 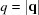 and on the exchange length 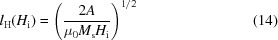
(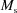: saturation magnetization; *A*: exchange-stiffness parameter; 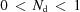: demagnetizing factor; 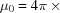 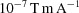). The θ dependence of 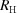 and 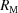 is essentially a consequence of the magnetodipolar interaction. Depending on the values of *q* and 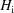, and on the ratio 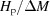, a variety of angular anisotropies may be seen on a two-dimensional detector (see *e.g.* Fig. 11 in §6.2 below) (Michels *et al.*, 2014 ▸; Michels, 2014 ▸).

**Figure 3:**
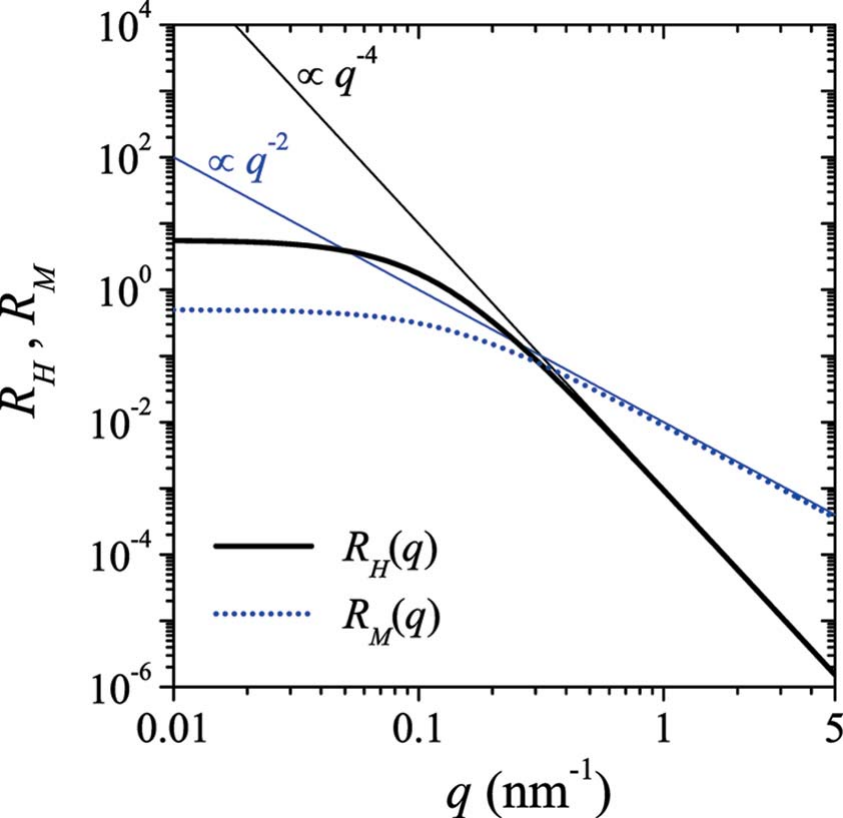
The dimensionless micromagnetic response functions 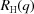 and 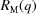 [equations (15) and (16)] at 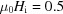
 T (log–log scale).

By assuming that the functions 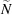, 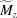 and *h* depend only on the magnitude 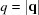 of the scattering vector, one can perform an azimuthal average of equation (4), *i.e.* 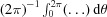. The resulting expressions for the response functions then read (see Fig. 3 ▸) 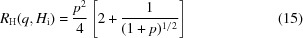
and 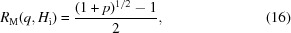
so that the azimuthally averaged total nuclear and magnetic unpolarized SANS cross section of a bulk ferromagnet can be written as 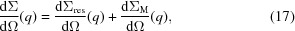
where 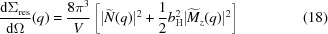
and 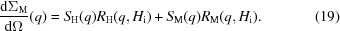



### 3.2. **k**_0_ ⊥ **H**_0_   

For the scattering geometry where the external magnetic field 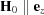 is parallel to the incident-beam direction 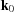 (compare Fig. 2*b*), the total unpolarized SANS cross section 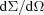 can be written as (Michels, 2014 ▸) 
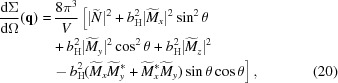
where 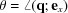. Using linearized micromagnetic theory, the azimuthally averaged version of equation (20) can be expressed as 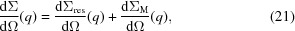
where the residual SANS cross section explicitly reads 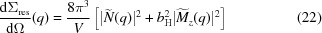
and the spin-misalignment SANS equals 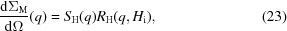
with 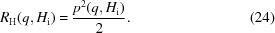


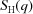
 is given by equation (7), and we note that in this geometry 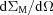 does not depend on 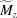 fluctuations and equals the expression for the single-phase material case (Weissmüller *et al.*, 1999 ▸), in other words, inhomogeneities in the saturation magnetization are (for 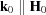) only contained in 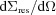 and not in 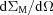.

## 4. Correlation function of the spin-misalignment SANS cross section   

Before addressing the magnetic correlation functions, we will briefly recall the corresponding well known results from nuclear SANS theory (Guinier & Fournet, 1955 ▸; Porod, 1982 ▸; Feigin & Svergun, 1987 ▸). The nuclear SANS cross section, 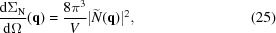
can be expressed in terms of the autocorrelation function 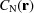 of the (excess) nuclear scattering length density 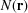 (in units of 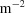) as 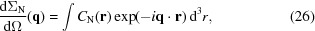
where 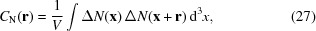
and 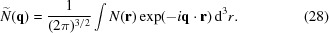
The function 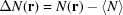 denotes the so-called excess scattering length density, where 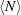 is the (constant) average scattering length density, which only gives a contribution to 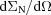 at 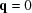. The back-transform of equation (26) is 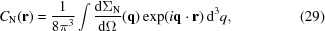
which for isotropic systems reduces to 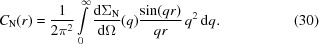
In analogy to the above formalism, one may define the autocorrelation function of the spin misalignment as (Michels *et al.*, 2003 ▸; Weissmüller *et al.*, 2004 ▸; Michels, 2010 ▸) 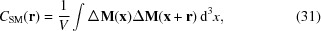
where 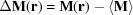 denotes the deviation of the local magnetization vector field 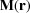 from the mean magnetization 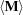. Alternatively, 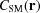 can be expressed as 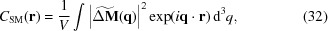
where 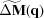 is the Fourier transform of 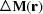. In the high-field limit, 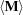 is nearly parallel to the applied magnetic field with 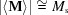, so that 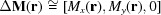 and 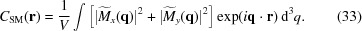
Note that in our theory of magnetic SANS (Honecker & Michels, 2013 ▸) the magnetization components 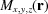 are all considered to be real valued.

Comparison of equations (27) and (31) reveals an important difference between nuclear and magnetic scattering [besides the fact that 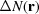 is a scalar and 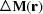 a vector quantity]: while the nuclear SANS cross section 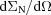 is directly proportional to the Fourier transform 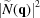 of 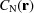, the function 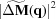 [being the Fourier transfrom of 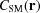] does not represent the experimentally measurable quantity 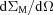, which, according to equations (3) and (20), is a weighted sum of the Cartesian Fourier components 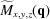 of the magnetization.

Therefore, we define the correlation function 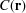 of the spin-misalignment SANS cross section as the Fourier transform of 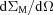, for which we have a theory, according to 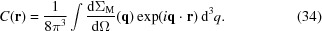
The normalized version of equation (34), 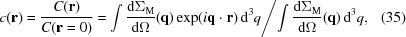
forms the basis for the calculations of the present work. We emphasize that the 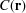 that is defined in this way is not an autocorrelation function, as are 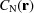 and 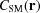. Likewise, the well known result that the evaluation of 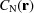 and 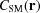 at the origin 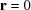 yields, respectively, the mean-squared density fluctuation (Porod invariant) and the mean-squared magnetization fluctuation does not pertain to 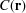; the integral of 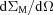 over reciprocal space does not provide an obvious invariant of the spin-misalignment SANS.

We remind the reader that 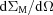 at a particular applied magnetic field 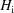 can be (approximately) obtained by subtracting the total nuclear and magnetic scattering at a saturating field from the measurement of the total 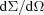 at the particular 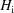.

The spin-misalignment SANS cross section for the perpendicular scattering geometry depends on both the magnitude *q* and the direction θ of the scattering vector 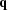 on the detector (see *e.g.* Fig. 11 in §6.2 below). The θ dependence of 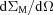 is a consequence of the magnetodipolar interaction – *via* the Fourier coefficients 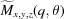 (Erokhin *et al.*, 2012 ▸; Honecker & Michels, 2013 ▸; Michels *et al.*, 2014 ▸) – and of the trigonometric functions which are explicitly contained in the cross section [equation (3)] and are due to the dipolar nature of the neutron–magnetic interaction. The final expression for the (azimuthally) θ-averaged 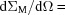 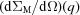 [equation (19)] contains the averages over these degrees of freedom. Since from a practical point of view it is easier to work with one-dimensional data, *i.e.* with 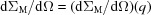, equation (35) may be simplified to 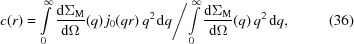
where 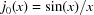 denotes the zeroth-order spherical Bessel function. Note that spherical Bessel functions are denoted with a lower-case ‘*j*’, whereas Bessel functions are represented with an upper-case ‘*J*’. Equation (36), which from now on is called the ‘one-dimensional’ correlation function of the spin-misalignment SANS cross section, has the same mathematical structure as the corresponding equation (30) for nuclear SANS.

Since for statistically isotropic bulk ferromagnets 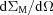 in the parallel scattering geometry is isotropic (independent of the angle θ) (Michels *et al.*, 2014 ▸), equation (36) also applies to 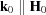.

In a SANS experiment, only the components of the momentum-transfer vector 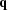 perpendicular to the incident-beam direction (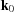) are effectively probed, which from a mathematical point of view means that the measured cross section already represents an average over the incident-beam direction. For 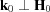 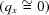, this implies that 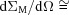 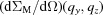, whereas 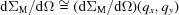 for 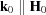 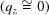 (compare Fig. 2 ▸). In §6.2 below, we will also study (for 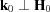) the case of anisotropic two-dimensional correlations by considering the following expression for 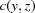 (Šaroun, 2000 ▸): 
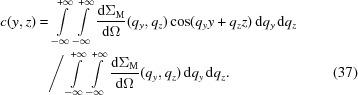
Because 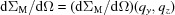, the 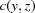 that is computed according to equation (37) represents a projection (average) of the three-dimensional correlation function 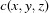 along the direction of the incident neutron beam (Fritz-Popovski, 2013 ▸, 2015 ▸).

Equation (37) can be transformed into polar coordinates, which results in 
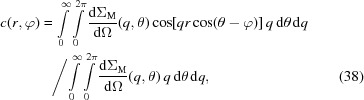
where the angle φ specifies the orientation of 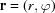 in the *yz* plane. By introducing the *n*th-order Bessel function (Watson, 1966 ▸), 
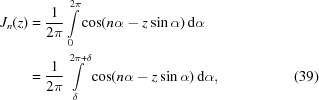
where *n* is an integer and the last equation is valid for any angle δ, we can obtain an average of 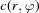 over all angles φ in the detector plane: 
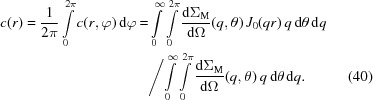
Since the integration with respect to the angle θ can be taken analytically [compare arguments leading to equations (15) and (16)], it follows that
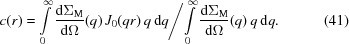
Equation (41) is called the averaged ‘two-dimensional’ correlation function of the spin-misalignment SANS cross section. Note that this expression differs from equation (36) which is obtained after three-dimensional integration of the θ-averaged 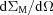.

In Appendix *A* [App appa], we provide a comparison between the autocorrelation function of the spin misalignment, 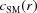, and the correlation functions of the spin-misalignment SANS cross sections, equations (36) and (41).

## 5. Models for *S*_H_ and *S*_M_   

In order to solve equation (36) [or equation (41)], we have to specify certain models for the anisotropy-field scattering function 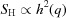 [equation (7)] and for the scattering function of the longitudinal magnetization 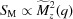 [equation (8)] in the expression for 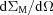. As outlined in §2, we consider a statistically isotropic nearly saturated bulk ferromagnet which exhibits (weak) spatial fluctuations of the saturation magnetization and the magnetic anisotropy field. For such a system, the functions 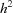 and 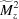 depend only on the magnitude *q* of the momentum-transfer vector 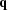. Furthermore, we assume a monodisperse scattering system and that both functions 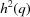 and 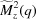 can be written as the product of the same single-particle form factor 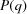 and structure factor 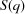 (Pedersen, 1997 ▸), *i.e.*

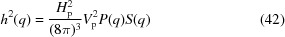
 and 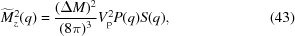
where 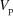 is the particle volume. Later on in the calculations, we will use (for illustration purposes) the Percus–Yevick hard-sphere structure factor for 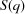 (Kinning & Thomas, 1984 ▸) and (unless stated otherwise) the sphere form factor for 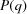, 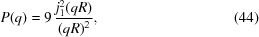
where 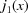 denotes the spherical Bessel function of first order. Any other particle form factor or structure factor may be straightforwardly implemented (see below). We also note that the characteristic structure sizes of 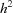 and 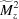 need not be identical; these are related, respectively, to the spatial extent of regions with uniform magnetic anisotropy field and saturation magnetization.

Under these assumptions (same size and shape), 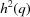 and 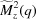 differ only by constant prefactors, *i.e.* the magnitude 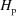 of the mean magnetic anisotropy field and the jump 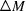 of the magnitude of the magnetization at internal interfaces. In fact, it is the ratio of 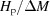 which determines the angular anisotropy and the asymptotic power-law dependence of 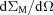 as well as the characteristic decay length of spin-misalignment fluctuations (Honecker & Michels, 2013 ▸).

In agreement with the assumption of a sharp interface in the nuclear (grain) microstructure (compare Fig. 1 ▸) both 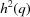 and 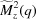 vary asymptotically as 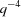. Together with the micromagnetic response functions which, respectively, vary as 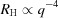 and 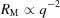 [compare equations (15) and (16), and see Fig. 3 ▸], this results in 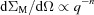 with *n* ranging between 6 and 8 (Honecker & Michels, 2013 ▸). We emphasize that other models for the anisotropy-field microstructure may result in different power-law exponents of 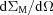; in particular, the 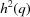 that are related to the long-range stress fields of dislocations are expected to give rise to asymptotic power laws that are different from the Porod exponent (Seeger, 1959 ▸; Heuser, 1994 ▸; Thomson *et al.*, 1999 ▸; Maxelon *et al.*, 2001 ▸; Long & Levine, 2005 ▸). This is, however, the subject of further investigations.

By inserting equations (42) and (43) into the θ-averaged spin-misalignment SANS cross sections [equations (19) and (23)], we can express the one-dimensional correlation functions of the spin-misalignment SANS cross section [equation (36)] as 
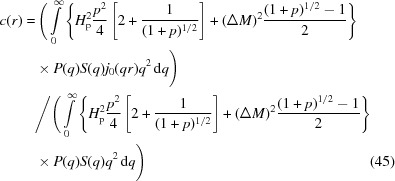
for 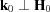 and 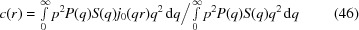
for 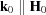. Note that 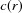 for the parallel geometry is [in contrast to 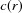 for the perpendicular case] independent of both 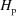 and 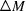; the dependence of 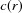 on the applied magnetic field 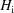 and on the magnetic interactions (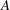, 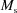) is contained in the function 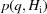 [compare equation (11)]. We also reemphasize that we have assumed that both Fourier coefficients 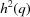 and 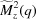 can be written as the product of the same form factor 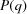 and structure factor 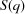; this assumption might be relaxed, *e.g.* when studying diffusion zones or core–shell-type nanoparticle structures with reduced surface magnetization (Heinemann *et al.*, 2000 ▸). The averaged two-dimensional correlation function [equation (41)] is obtained by making the corresponding replacements in equations (45) and (46).

## 6. Results and discussion   

The following materials parameters were used in the calculations: saturation magnetization 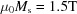, exchange-stiffness constant 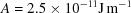 and 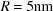 for the particle radius in the sphere form factor 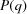 [equation (44)].

### 6.1. One-dimensional correlation functions   

All results in this section are obtained by numerical integration of equations (45) and (46), which are based on the one-dimensional correlation function equation (36). In the first set of calculations, we concentrate on the dependence of the correlation functions on the applied magnetic field 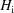, scattering geometry (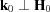 and 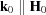), ratio 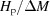, single-particle form factor 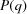 and structure factor 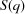.

**Figure 4:**
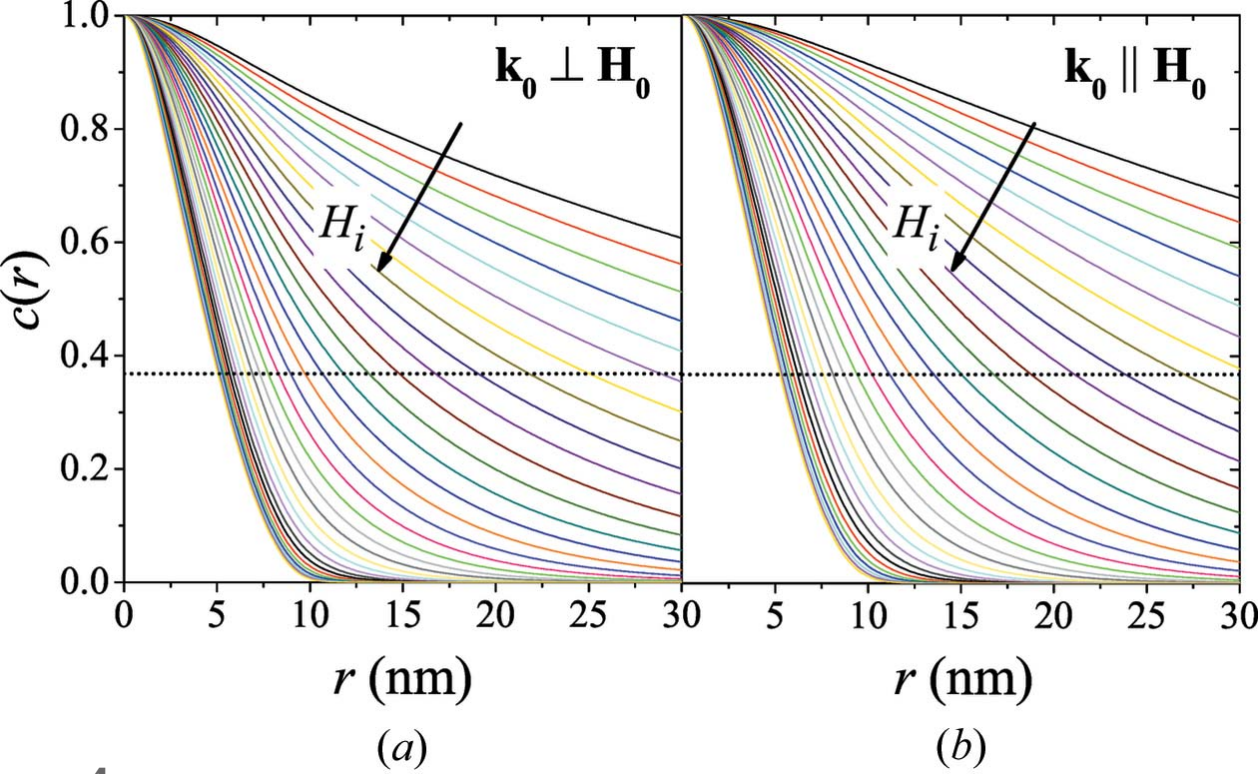
Normalized correlation functions 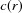 of the spin-misalignment SANS cross section at several applied-field values 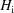 for (*a*) 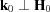 and (*b*) 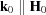. 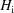 increases, respectively, from 0.01 to 100 T on a logarithmic scale, *i.e.* 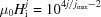
 T, where 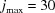 and 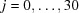 [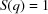; 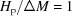]; the arrows specify the direction of increasing 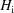. Dotted horizontal lines in (*a*) and (*b*): 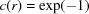.

Fig. 4 ▸ displays the results for 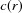 at several values of 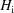 and for both scattering geometries, assuming a dilute scattering system [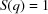] and 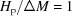. The dotted horizontal lines indicate the value of the correlation length 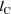 of the spin misalignment, which can be taken as a measure of the size of inhomogeneously magnetized regions around defects. 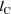 is defined as the 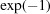 decay length, *i.e.* 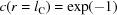. Note, however, that this definition does not imply that the correlations decay exponentially. In fact, it is readily verified that the spin-misalignment correlations that are investigated in this study do not decay exponentially. We would also like to mention that an alternative route to extracting a spin-misalignment length may be realized by the computation of moments of the correlation function; for instance, for exponentially decaying 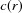 the above definition and 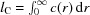 are equivalent.

**Figure 5:**
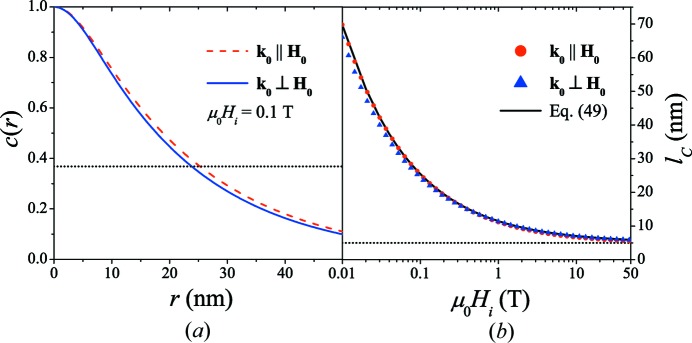
(*a*) Comparison of the 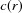 for the two scattering geometries [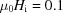
 T; 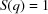; 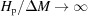]. Dotted horizontal line: 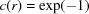. (*b*) Comparison of the field dependence of the spin-misalignment correlation length 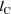 for the two scattering geometries [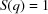; 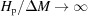] (log–linear scale). Solid line: equation (49). Dotted horizontal line: 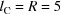
 nm.

**Figure 6:**
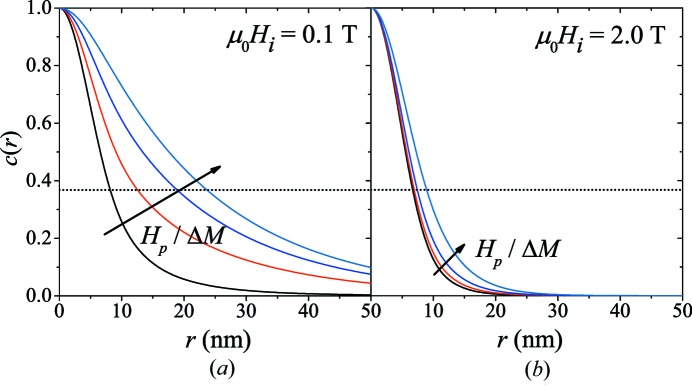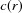 for several values of the ratio 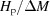 at (*a*) 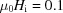
 T and (*b*) 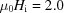
 T [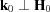; 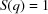]. 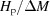 values: 0.004, 0.4, 0.8, 4; the arrows specify the direction of increasing 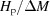; for larger values of 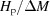, 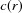 remains effectively unchanged. Dotted horizontal lines in (*a*) and (*b*): 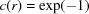.

Increasing 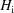 results in both scattering geometries in the suppression of transverse spin-misalignment fluctuations and in a concomitant reduction of the 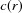 and reduced 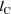 values. At small fields, 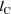 may take on values of the order of 100 nm, which decrease to values of the order of the assumed particle size, here 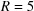
 nm, for fields larger than a few tesla [see also dotted horizontal line in Fig. 5 ▸(*b*)]. For the chosen limiting case of 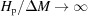, the difference between the 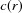 and the 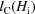 in the two scattering geometries is only minor (see Fig. 5 ▸). However, noting that 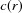 in the parallel geometry is independent of 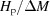 and with reference to Figs. 6 ▸ and 7 ▸, we note that this difference increases with decreasing value of 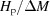.

Within the framework of our micromagnetic SANS theory of bulk ferromagnets (Honecker *et al.*, 2013 ▸; Metlov & Michels, 2015 ▸), the magnetic microstructure in real space, 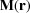, corresponds to a complicated convolution product between the magnetic anisotropy-field microstructure and micromagnetic functions. As a result, smoothly varying magnetization profiles are at the origin of the related spin-misalignment scattering. In agreement with the absence of a sharp interface in the magnetic microstructure (compare Fig. 1 ▸ *b*), we note that the correlation functions of bulk ferromagnets enter the origin 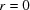 with zero slope (Bick *et al.*, 2013 ▸), so that 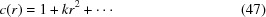
for 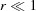 (where *k* is a constant). This observation may be compared to the well known result for nuclear particle scattering, where (for isolated uniform particles) the first derivative of 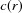 evaluated at 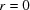 is related to the particle surface. In particular, for small *r*, the correlation function can be expanded as (Porod, 1982 ▸) 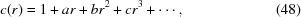
where the ‘differential’ parameters 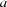, 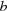, 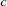 are related to the size and shape of the particle; for example, for a uniform sphere one finds 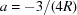, 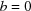 and 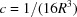.

The effect of the ratio 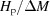 on the correlation functions and on the 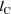 values is shown in Figs. 6 ▸ and 7 ▸ [for 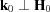 and 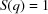]. Perturbations in the spin microstructure that are dominated by fluctuations of the magnetic anisotropy field (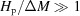) decay on a larger length scale than magneto­statically dominated (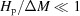) perturbations.

For soft magnets (with low crystalline anisotropy), the following relation for 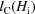 has previously been suggested (Michels, 2014 ▸):
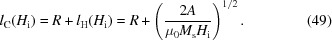
Equation (49) provides an excellent description of the field-dependent correlations [solid lines in Figs. 5 ▸(*b*) and 7 ▸ with 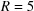
 nm, 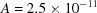
 J m^−1^ and 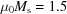
 T]. At large fields, when the spin-misalignment SANS cross section is small and the exchange length 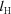 takes on values of a few nanometres, 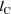 reflects, irrespective of 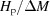, the size of the (in this case spherical) defect.

**Figure 8:**
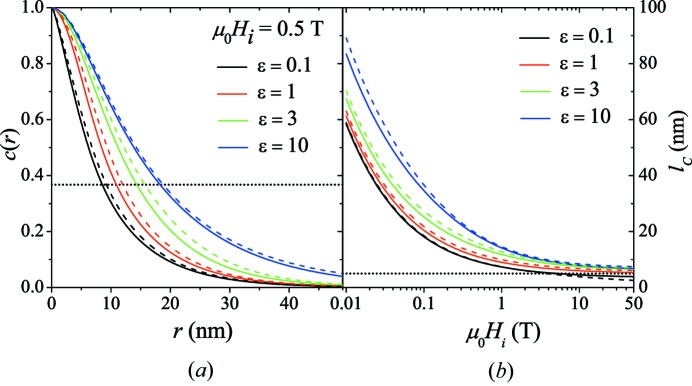
Effect of particle form factor on the correlation function and correlation length. (*a*) 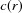 at 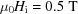 and for several particle form factors. Solid lines: form factor of ellipsoid of revolution (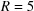
 nm) with ∊ decreasing from top to bottom (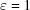 corresponds to the sphere form factor). Dashed lines: cylinder form factor with 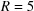
 nm and 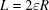 [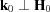; 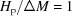; 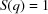]. Dotted horizontal line: 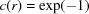. (*b*) Corresponding 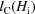 (log–linear scale). Dotted horizontal line: 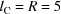
 nm.

For the perpendicular scattering geometry, Fig. 8 ▸ displays (for 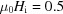
 T) the correlation function for different single-particle form factors 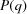, ignoring interparticle interactions [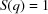]. In addition to the sphere form factor [equation (44)], we have used in the expressions for 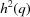 and 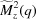 the cylinder form factor (radius: *R*; length: *L*) (Pedersen, 1997 ▸), 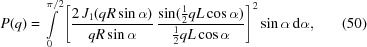
and the form factor of an ellipsoid of revolution (semi-axes: 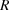, 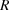, 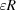), 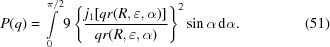


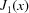
 denotes the first-order Bessel function, 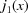 is the first-order spherical Bessel function and 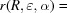 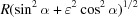. Note that equation (51) reduces to the sphere form factor for 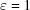. Besides the cylinder and ellipsoid of revolution form factor we have also used other form factors (data not shown); the above form factors were chosen because they allow one to investigate different limiting cases (from thin circular discs to elongated spheroids and elongated thin rods). Examples for bulk magnetic materials with elongated cylindrically or elliptically shaped precipitates are alnico magnets (Zhou *et al.*, 2014 ▸), which are nanostructured alloys composed of Fe, Al, Ni and Co.

It is seen in Fig. 8 ▸ that for a given form factor the shape of the correlation function and the value of the correlation length depend (as expected) on the particle dimensions. Isotropically distributed cylinders (dashed lines) with a radius equal to the radius of the ellipsoid of revolution and a length 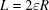 result in nearly the same (slightly larger) correlation functions as the ellipsoid of revolution. 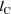 at large fields appears to be related to the smallest dimension of the particle, although the precise dependency of 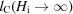 on the particle dimensions is not clear to us. Note also that for the case of very thin discs and oblate spheroids (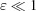) the correlation function still approaches the origin with zero slope (which becomes visible only for small *r*).

**Figure 9:**
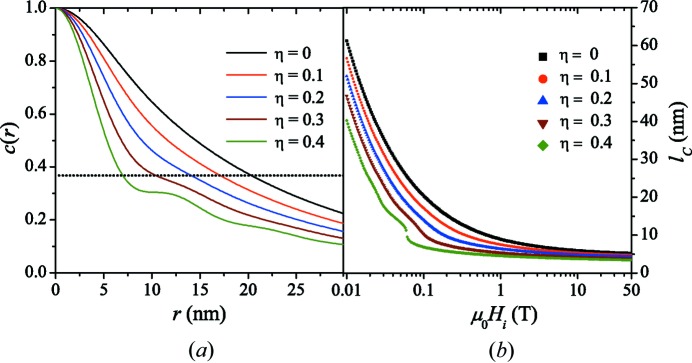
Effect of hard-sphere volume fraction η on the correlation function and correlation length. (*a*) 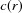 at 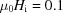
 T and for several values of η increasing from top to bottom (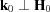; 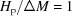). Dotted horizontal line: 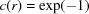. (*b*) Corresponding 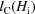 (log–linear scale).

Finally, Fig. 9 ▸ illustrates the effect of interparticle interactions on the correlation function (Fig. 9 ▸ *a*) and correlation length (Fig. 9 ▸ *b*). In order to model the effect of dense packing, we have used the Percus–Yevick hard-sphere structure factor for 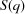 (Kinning & Thomas, 1984 ▸) in equations (45) and (46) and, as before, the sphere form factor for 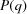. Note also that the hard-sphere interaction radius 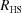 in 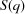 was set equal to the sphere radius *R*.

It is clearly seen that with increasing particle volume fraction η the range of the correlations decreases. However, the characteristic features of the structure factor only become visible at relatively large values of η (above about 20%), while at the lower end of η values both 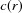 and 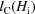 are smoothly decaying functions. Furthermore, we note that with increasing η, *i.e.* with increasing interparticle interactions, we progressively introduce, in addition to the original (diffuse) spin-misalignment length 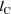, a second structural correlation length into the system (compare *e.g.* the hump in 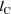 at around 50 mT for 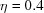).

**Figure 10:**
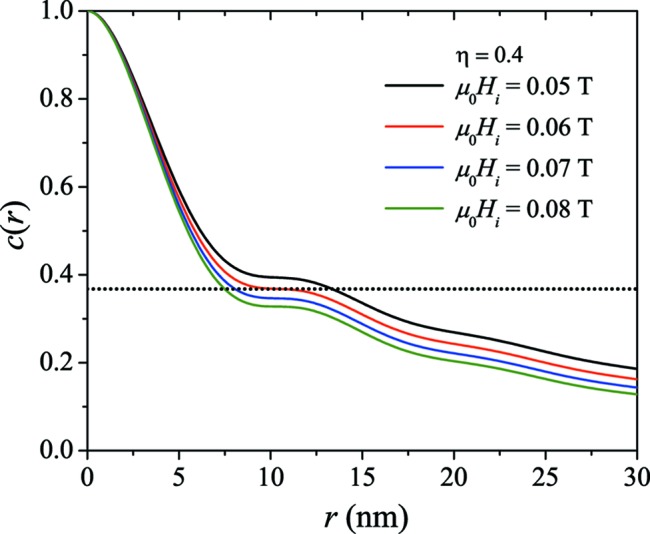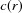 for 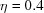 and for several values of the applied magnetic field 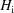 increasing from top to bottom (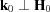; 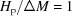). Dotted horizontal line: 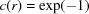.

The field dependence of this feature is depicted in Fig. 10 ▸, where we show 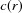 for several 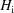 and for 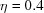; here, we see that slight changes in 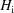 result in relatively large jumps in 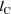 [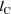
(0.08 T) 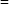 7.7 nm and 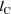
(0.05 T) 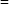 13.8 nm]. This is an artifact which is clearly related to the strong structural correlations, and the determined correlation length now represents a field-dependent (unknown) average over the structural and the magnetic spin-misalignment correlation lengths. We note that by using other definitions for 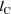, for instance in terms of some integral weight over 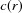, the position of the artifact on the 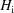 axis may be different but the effect of 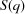 will still become visible.

### 6.2. Two-dimensional correlation functions   

**Figure 11:**
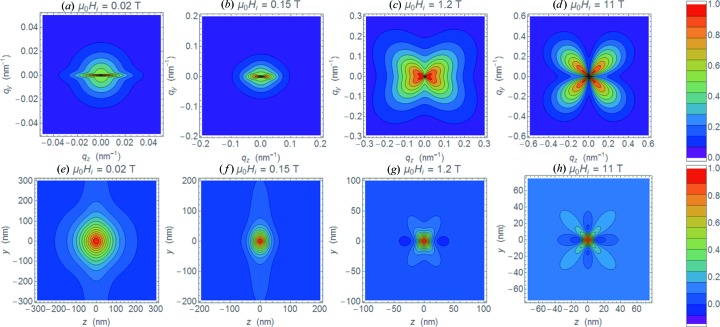
(*a*)–(*d*) Contour plots of normalized 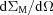 [equation (6)] at applied magnetic fields as indicated (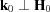; 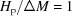; 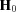 is horizontal). For 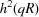 and 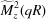, we used the form factor of a sphere with a radius of 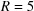
 nm [equation (44); 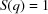]. (*e*)–(*h*) Corresponding two-dimensional correlation functions 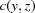, which were computed according to equation (37) (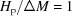).

Since the spin-misalignment SANS cross section is highly anisotropic for 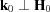, the corresponding correlation function may also be anisotropic. We reemphasize that the angular θ dependence of 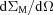 is a consequence of the trigonometric functions in the cross section (which are due to the dipolar neutron–magnetic interaction) and of the θ dependence of the magnetization Fourier coefficients 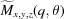 (which is due to the internal magnetostatic interaction) (Michels, 2014 ▸). Figs. 11 ▸(*a*)–11 ▸(*d*) show 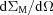 [equation (6)] at selected applied magnetic fields [and for 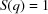]. The change in the angular anisotropy that becomes visible in Figs. 11 ▸(*a*)–11 ▸(*d*), from a spike-type anisotropy at low fields (*a*) to a clover-leaf-shaped anisotropy at large fields (*d*), is related to the field dependence of the Fourier coefficients and demonstrates that different terms in the response functions [equations (9) and (10)] dominate in different field regimes. For instance, the spike anisotropy (Fig. 11 ▸ *a*) was recently observed in an isotropic sintered Nd–Fe–B magnet (Périgo *et al.*, 2014 ▸); it is related to magnetostatic terms 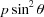 in the denominator of the response functions.

The corresponding two-dimensional correlation functions, computed according to equation (37), are displayed in Figs. 11 ▸(*e*)–11 ▸(*h*), where we plot the 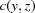 at the same fields as the 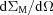. While the spin-misalignment SANS cross section at small fields (Figs. 11 ▸ *a* and 11 ▸ *b*) is enhanced parallel to the applied-field direction, the correlation function exhibits maxima in the direction perpendicular to the field; the range of the correlations extends to several hundreds of nanometres (Figs. 11 ▸ *e* and 11 ▸ *f*). Increasing the field results in the suppression of the correlations. At the largest field 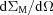 possesses a nearly fourfold anisotropy with maxima along the detector diagonals and minima along the horizontal and vertical axes (Fig. 11 ▸ *d*), which translate into the corresponding extrema in 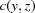 (Fig. 11 ▸ *h*).

**Figure 12:**
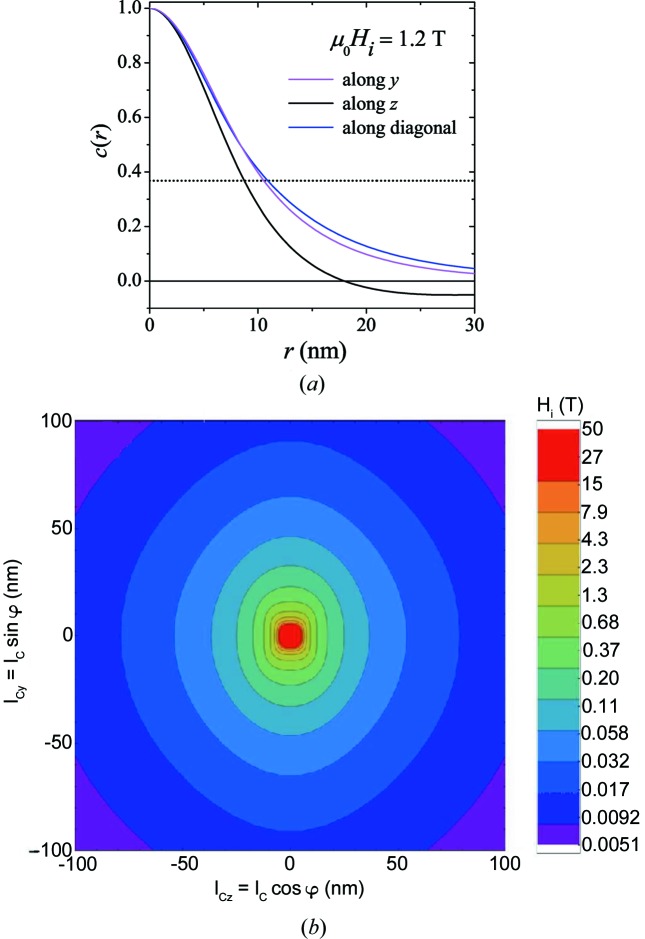
(*a*) 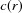 along different real-space directions [same parameters as in Fig. 11 ▸(*g*)]. Dotted horizontal line: 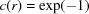. (*b*) Contour plot revealing the in-plane (φ) variation of 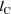 for several values of the applied magnetic field 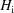. Logarithmic color scale for the field is used.

In Fig. 12 ▸(*a*), we depict the correlation function along different directions: while the correlation length at 1.2 T varies only relatively little with direction (from 8.8 to 10.9 nm), the functional dependencies of the 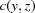 are significantly different, with the correlation function along the horizontal **z** direction becoming negative at 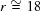
 nm; the curves in Fig. 12 ▸(*a*) were obtained by solving equation (38) for 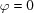, 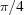, 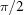. In nuclear SANS, negative values of the distance distribution function 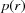 are attributed to distances that connect regions with opposite sign of the scattering length density more frequently than regions with the same sign (Glatter & Kratky, 1982 ▸). However, for magnetic SANS, such an easily accessible interpretation of the correlation function 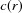 of the spin-misalignment SANS cross section in terms of a specific magnetization distribution is not straightforward; this is mainly related to the (above mentioned) fact that 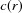 does not directly represent the correlations in the magnetic microstructure (as does 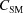), but also includes the magnetodipolar interaction of the neutrons with the sample (*via* the trigonometric functions and the cross term in the cross section). The anisotropy of the correlations is further depicted in Fig. 12 ▸(*b*), where we show a contour plot for several values of 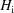. This graph reveals a relatively weak anisotropy of 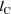. At small fields, the correlations along the vertical (**y**) direction decay on a larger length scale than along the horizontal (**z**) direction; with increasing field, the anisotropy becomes less pronounced.

**Figure 13:**
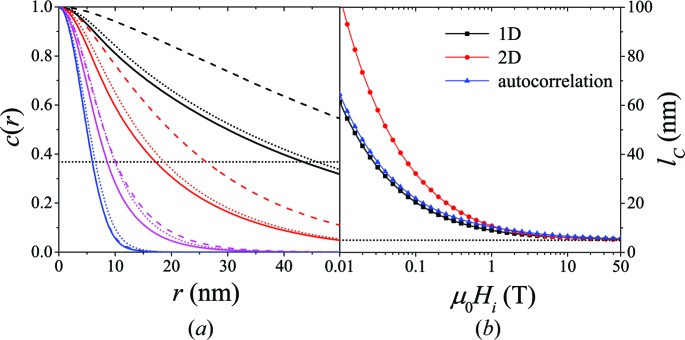
(*a*) Comparison between the one-dimensional [equation (36); solid lines] and the averaged two-dimensional [equation (41); dashed lines] correlation functions of the spin-misalignment SANS cross section and the autocorrelation function of the spin misalignment (dotted lines, see Appendix *A* [App appa]) [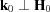; 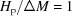; 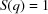]. 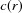 at selected 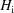; values of 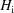 (in T) increasing from top to bottom: 0.02, 0.15, 1.2, 11. Dotted horizontal line: 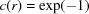. (*b*) Corresponding 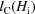 (log–linear scale) (solid lines are guides to the eyes). Dotted horizontal line: 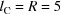
 nm.

Fig. 13 ▸ compares (for 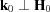) the results for the one-dimensional [equation (36)] and the averaged two-dimensional [equation (41)] correlation functions of the spin-misalignment SANS. We recall that the former is obtained by three-dimensional integration of the azimuthally averaged 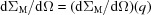, and the latter by two-dimensional integration of 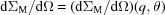 (compare §4). At small fields, the results for 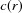 and 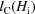 differ considerably, whereas for 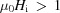
 T both equations yield almost the same correlation lengths.

The question may arise as to which one of these correlation functions should be used in order to analyze experimental data. From an experimental point of view, the averaged two-dimensional equation (41) reflects the data-analysis procedure, namely that the measured 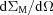 is a function of only two independent components of the scattering vector; in fact, elastic scattering in the small-angle approximation only probes correlations in the directions perpendicular to the incident beam. Reconstruction (from experimental 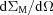) of the one-dimensional 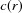 (which is an orientation average of the three-dimensional correlation function) is always an extrapolation.

### 6.3. Comparison with experimental data   

**Figure 14:**
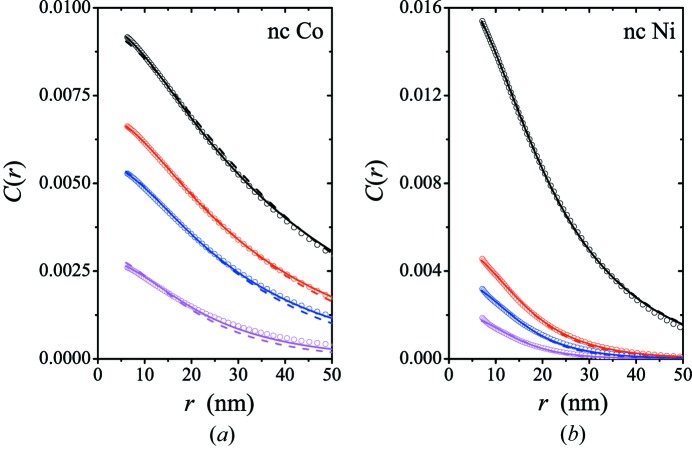
Comparison between experimental and theoretical data. (Open circles) Correlation functions of the spin-misalignment SANS cross section of (*a*) nanocrystalline Co and (*b*) nanocrystalline Ni with average crystallite sizes of 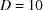
 nm (Co) and 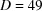
 nm (Ni) (Weissmüller *et al.*, 2001 ▸). 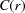 data are taken from Michels *et al.* (2003 ▸). Solid lines: fit based on equation (36); dashed lines: fit based on equation (41). Values of the internal magnetic field 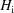 (in mT) from top to bottom, respectively: (*a*) 54, 80, 107, 243; (*b*) 190, 570, 800, 1240. In both analyses, we have used the sphere form factor for 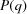 and 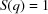.

In order to test our magnetic SANS theory, we depict in Fig. 14 ▸ a comparison between experiment and theory; in particular, we have fitted equations (36) and (41) [using, in each case, equation (19) for 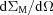] to experimental data for the correlation function of the spin-misalignment SANS cross section of nanocrystalline Co and Ni (Michels *et al.*, 2003 ▸). These 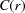 data have previously been analyzed by Michels & Bick (2013 ▸) using a simple approach based on the autocorrelation function of the spin misalignment, neglecting terms due to spatial fluctuations of the saturation magnetization. Such contributions are included in the present theory *via* the term 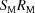 in equation (19). The nanocrystalline Co and Ni samples constitute fully dense polycrystalline metals with average crystallite sizes of 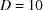
 nm (Co) and 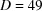
 nm (Ni) (Weissmüller *et al.*, 2001 ▸). The experimental SANS data of both samples were recorded between 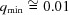
 nm^−1^ and 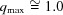
 nm^−1^. The correlation functions were then obtained by direct Fourier transformation according to equation (36), so that this expression should actually also be used for the data analysis. Nevertheless, we have also employed the two-dimensional equation (41) for fitting the experimental 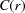 data, which is motivated by the fact that for larger applied fields the difference between the two correlation functions is only minor (compare Fig. 13 ▸). In the following discussion, one should therefore keep in mind that for the analysis of this particular 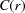 data set equation (36) represents the proper theoretical model.

In the fitting procedure, the integrals in equations (36) and (41) were approximated by discrete sums, where the upper integration limit of ‘
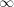
’ was taken as 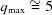
–10 nm^−1^ and the typical *q* resolution was set to 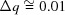
–0.02 nm^−1^. The resulting expressions were fitted by means of a nonlinear (Levenberg–Marquardt) fitting routine to the experimental 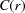 data. We have treated the exchange-stiffness constant *A*, the ratio 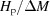 and *R* as global fit parameters. Since we work with unnormalized 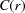 data, we have introduced field-dependent local scaling constants 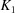, 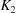, 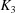 and 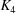 (one for each data set); 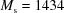
 kA m^−1^ for Co and 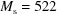
 kA m^−1^ for Ni were held constant. Since the experimental SANS data [*e.g.* Fig. 1 of Michels *et al.* (2003 ▸)] do not give a visible indication of a strong impact of dense packing, we have for simplicity decided to set 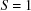. The results for the global fit parameters are summarized in Table 1 ▸. The data analysis was restricted to *r* values below about 50 nm and to fields larger than 50 mT, where the magnetization of both samples approaches saturation (Weissmüller *et al.*, 2001 ▸).

As is seen in Fig. 14 ▸ (solid and dashed lines), both equations provide a reasonable global description of the field-dependent correlations. The obtained values for the anisotropy-field radii *R* of both materials are in the range 8–13 nm, slightly smaller than the ones estimated previously (Michels & Bick, 2013 ▸). The parameter *R* characterizes the length scale over which the magnetic anisotropy field 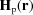 is uniform; for single-crystal grains, *R* is sensibly related to the average crystallite size (compare Fig. 1 ▸ *a*). Therefore, the finding 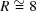
–10  nm for Co suggests that the magnetic anisotropy field is approximately homogeneous on a length scale of the order of the average grain size of 10 nm, whereas for Ni nonuniformities in 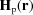 exist on a scale smaller than the average crystallite size of 49 nm, presumably related to twin faults or to the defect cores of grain boundaries (Michels *et al.*, 2003 ▸). While the obtained values for the exchange-stiffness constant of Ni (using both equations) are larger by a factor of about two than the ones reported in the literature (Kronmüller & Fähnle, 2003 ▸), the *A* value for Co using equation (41) agrees excellently with literature data and with the result of our previous SANS data analysis (in Fourier space) (Michels *et al.*, 2003 ▸). Values for the ratio of 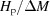 have not been determined previously for these materials, but our results suggest [except for the case of Ni using equation (41)] that perturbations in the spin microstructure due to spatially fluctuating magnetic anisotropy fields dominate over magnetostatic fluctuations. This might be expected, since in single-phase ferromagnets variations in 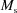 are relatively small, compared to *e.g.* nanocomposites (Michels *et al.*, 2006 ▸). Overall, the good agreement between experiment and theory suggests that equation (36) may be used for the analysis of real-space correlations of bulk magnetic materials; equation (41) may also be employed for the analysis of experimental data, provided that the original 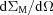 has been Fourier transformed according to equation (41).

## 7. Summary and conclusion   

On the basis of a recent micromagnetic theory for the magnetic SANS cross section of inhomogeneous bulk ferromagnets, we have studied the corresponding magnetic field-dependent spin-misalignment correlations in real space. The correlation function 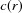 of the spin-misalignment SANS cross section depends on the applied magnetic field and, for 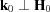, on the ratio of magnetic anisotropy field strength 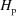 to magnetization jump 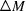 at internal interfaces. Additional degrees of freedom in 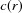 relate to the particle (anisotropy-field) form factor or to the inclusion of interparticle correlations *via* a structure factor. The result for 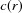 (for 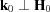) [equation (45)] demonstrates a strong impact of 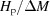 on the shape and range of the correlations: magnetostatically dominated correlations (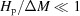) decay on a rather short length scale, whereas anisotropy-field-dominated correlations (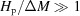) are characterized by a long-range decay, which is reasonably described by equation (49). The difference between the correlation functions in the two scattering geometries (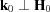 and 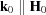) increases with decreasing ratio of 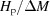. The correlation functions do not decay exponentially and approach the origin with zero slope; as far as equation (36) is concerned, this is consistent with the absence of a sharp interface in the magnetic microstructure. Experimental data for the correlation function of the spin-misalignment SANS cross section of nanocrystalline Co and Ni have been successfully analyzed using the here presented theoretical expressions. It would also be of interest to employ the present approach for studying long-range magnetic correlations, as accessible on a USANS instrument (Jericha *et al.*, 2013 ▸), or the magnetic microstructure of state-of-the-art nanocrystalline NdFeB-based permanent magnets (Bick *et al.*, 2013 ▸; Yano *et al.*, 2014 ▸; Périgo *et al.*, 2015 ▸; Saito *et al.*, 2015 ▸).

## Figures and Tables

**Figure 7:**
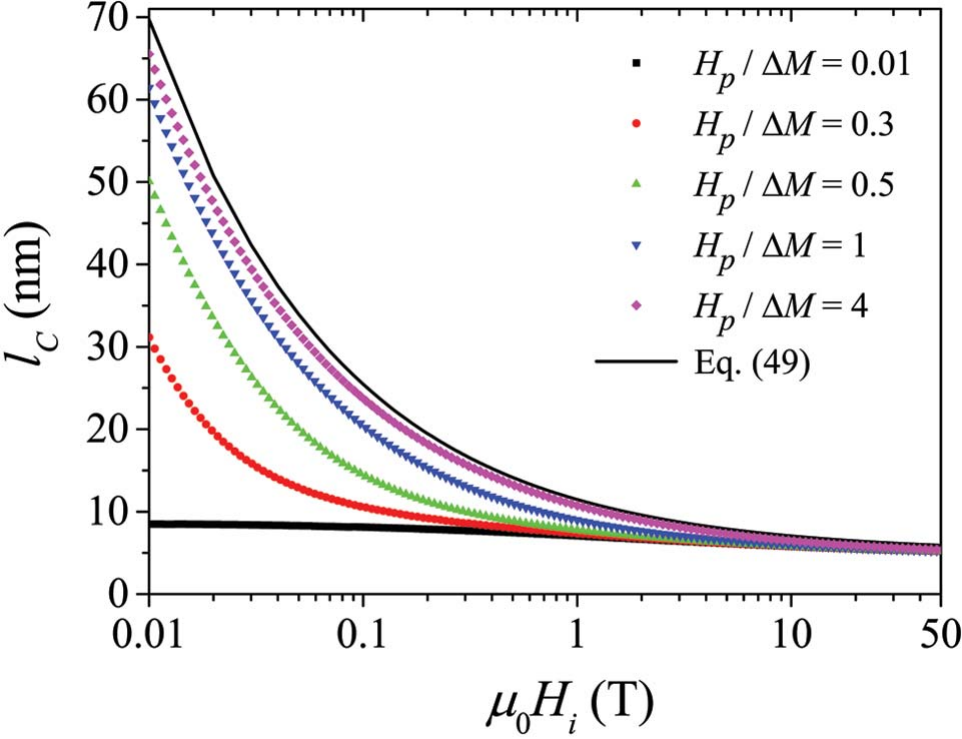
Field dependence of the spin-misalignment correlation length 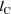 for different values of 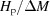 [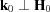; 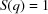] (log–linear scale). Solid line: equation (49).

**Table 1:** Results for the global fit parameters *A*, 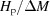 and *R* obtained by fitting equations (36) and (41) to the correlation functions of nanocrystalline Co and Ni displayed in Fig. 14 ▸

	Co [equation (36)]	Co [equation (41)]	Ni [equation (36)]	Ni [equation (41)]
*A* (pJ m 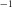 )	54.6 (6)	29.1 (6)	15.1 (1)	13.7 (4)
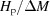	13.4 (0)	4.0 (1)	5.6 (0)	0.5 (1)
*R* (nm)	10.2 (1)	8.2 (6)	9.9 (1)	13.0 (1)

## Appendix A Autocorrelation function of the spin misalignment

In the high-field limit and for a general orientation of the wavevector , the solution, in Fourier space, of the linearized balance-of-torques equation (1) can be written as (Honecker & Michels, 2013 ▸)

For  or , one obtains the expressions for  and , respectively, which enter the equations for the perpendicular or the parallel SANS cross sections [equations (3) and (20)]. Averaging the expression for  over the orientation (angle β) of the magnetic anisotropy-field Fourier coefficient,

results in

where  and  is given by equation (11). Inserting this function into the normalized version of equation (33) allows us to obtain the autocorrelation function of the spin misalignment, , by three-dimensional integration. Fig. 13 ▸ displays  and  and compares the results with the correlation functions of the spin-misalignment SANS cross section, equations (36) and (41).
